# Differences in incidence and survival to childhood cancer between rural and urban areas in Castilla y León, Spain (2003–2014): A Strobe-compliant study

**DOI:** 10.1097/MD.0000000000012797

**Published:** 2018-10-12

**Authors:** Hermenegildo González García, Rebeca Garrote Molpeceres, Elena Urbaneja Rodríguez, Pilar Gutiérrez Meléndez, Raquel Herráiz Cristóbal, María Asunción Pino Vázquez

**Affiliations:** a Division of Haematology/Oncology, Department of Pediatrics, Valladolid Clinic University Hospital, C/ Ramón y Cajal; b Health Public Observatory of Junta de Castilla y León, Consejería de Sanidad, Paseo de Zorrilla, Valladolid, Spain.

**Keywords:** childhood cancer, incidence, population-based registry, survival, urban and rural areas

## Abstract

The aim of this study is to describe childhood cancer incidence and survival in Castilla y León (Spain) for the period 2003 to 2014 and to explore differences between rural and urban areas.

We made a cohort study in the childhood population of our region for the period of years referred before. Age-adjusted incidence rates to the world standard population (ASRw) were calculated by direct method, and their comparisons were made using incidence rate rations. Survival proportions were calculated by Kaplan-Meier method and their comparisons with log-rank test. The median childhood population less than 15 years old was 296,776 children. A total of 615 cases were recorded from the population-based Childhood Cancer Registry, including all malignant and benign tumors of the central nervous system.

Age-standardized incidence rates for all cancers were 176.6 per million. Leukemia incidence rates were highest in rural areas (51.08/million) than in urban areas (33.65/million; *P* = .018), and by age groups; these differences only remained at age 0 to 4 years with higher rural leukemia incidence (67.13/million) than in urban areas (39.32/million; *P* = .05). There were no statistically significant differences between rural and urban areas for lymphomas, central nervous system, and all other malignant solid tumors grouped. The 5-year overall survival rate for all patients was 84%, similar to other developed countries, with greater survival in rural areas (88%) compared with urban areas (80%; *P* = .033). The analysis by tumor groups showed a greater survival rate in rural areas for all the groups, although these differences only reached statistical significance in the group of leukemias, with a survival rate of 90% for rural areas compared with 76% for urban areas (*P* = .01). Analyzing survival rate by age groups in leukemias only significant survival differences at 10 to 14 years were encountered.

We found a higher incidence of leukemia in girls, mainly in rural areas, and also a better survival rate in children diagnosed with leukemia belonging to this population area. Future studies that analyze these facts in similar populations can help us clarify what genetic, epigenetic and environmental factors influence our population and are responsible for these findings.

## 1 Introduction

Childhood^[1]^ cancer is a pathology of great social-health relevance. The overall incidence rate for 0 to 14 years old is 140.6 per million person-years, age-adjusted using the world standard population (ASRw), yet it varies considerably between regions, and also between racial and ethnic groups.^[1]^ Overall ASRw varies from more than 150 per million person-years in some subpopulations of North America, Europe, especially in Southern Europe, and in Oceania, to less than 100 per million in sub-Saharan Africa, for Native American children in the USA, and in South Asia (India).

There^[2–4]^ are many research groups dedicated to the etiological study of childhood cancer in the world, trying to detect avoidable risk factors to establish preventive measures.^[5,6]^ Classical model of childhood cancer origin is based in the presence of a chromosomal translocation which needs a second genetic mutation to originate a cancer cell. These cells acquire their oncogenic potential through different stages of training.^[7]^ It is known that almost half of all childhood tumors have a prenatal origin, in which some genes involved in embryonic development are involved.^[8]^ Actually new models of childhood cancer development defend the confluence of 3 factors: genetic mutations,^[9]^ epigenetic deregulation,^[10]^ and environmental risk factors.^[11]^ Taking into account possible differences in exposure to environmental agents between individuals living in urban and rural areas that may induce genetic and epigenetic changes,^[12]^ it might be interesting to study the incidence and survival of childhood cancer among these areas. If there are differences in it, these would aid to provide potentially useful biomarkers for cancer diagnosis, not only in our region but also in the world.

Leukemia,^[1]^ central nervous system (CNS) tumors, and lymphomas are the childhood cancer types more frequent in this order. Leukemia is the most common type of them and accounts for 25% to 35% of all cases.^[1,13]^ For this reason, there are more studies that seem to explain its origin. Acute leukemia (AL), including acute lymphoblastic leukemia (ALL) (78%) and acute myeloblastic leukemia (AML) (16%), is predominant in childhood.^[14,15]^ Although the etiology of childhood leukemia remains unknown, it is believed that both genetic and environmental factors are involved.^[16]^ The genetic influence is evidenced by factors such as the higher rates in Hispanic children in the USA and by association with some genetic syndromes.^[1,12,15]^ Both childhood leukemias have increased over the past 4 decades (at an average rate of 0.7% per year), and higher incidence differences by world geographical region indicate that the origins of childhood cancer like leukemia are not only influenced by genetic factors but also by epigenetic and environmental factors.^[12,15]^ Ionizing radiation has been established as 1 causal risk factor, whereas other less determinant environmental exposure factors include ambient exposure early in life to traffic air pollution, paternal exposure before conception, and maternal exposure before conception or during pregnancy to solvents, pesticides, and tobacco smoke.

The objectives of this study are to describe the incidence and survival to childhood cancer diagnosed between 2003 and 2014 in Castilla y León (CL, Spain) and to investigate differences by population medium (rural and urban). Based on explains before, our main hypothesis was that there might exist differences in childhood cancer incidence and/or survival between urban and rural areas. We will focus our study on the analysis of leukemia due to its frequency in childhood. For proving the hypothesis, we made an observational cohort study in our childhood region population.

## 2 Materials and methods

### 2.1 Study population

The region of CL is a southern European region located in central Spain, with a total population of 2,478,079 inhabitants in 2014.^[16]^ It covers an area of 94,227 km^2^ and has a low population density of 25.97 inhabitants/km^2^, 2248 municipalities, and 56% urban population. Only 1 city has over 300,000 inhabitants, whereas a further 3 have over 100,000 inhabitants and a further 11 towns have over 20,000 inhabitants.

Study population included all children under 15 years old with a first diagnosis of cancer, including benign tumors of CNS, who inhabited our region during the study period (since January 1, 2003 to December 31, 2014).

The study period was chosen because it was more than 10 years, and had complete and updated patient information, which allowed us to obtain a sufficient number of subjects in each subgroup to realize analyses of incidence and 3 and 5-year overall survival analyses and make further comparisons.

The reference population (risk population) came from the 2003 to 2014 data provided by the Spanish National Institute of Statistics. The total population (<15 years) was 285,419 in 2003 and 301,768 in 2014, and average population during this period was 296,776 to 131,762 in rural and 165,014 in urban areas.^[17]^ According to the specific demographic characteristics of CL, an urban area was defined as a population of more than 20,000 inhabitants and rural areas as those with less than 20,000 inhabitants according to the spatial planning plan of CL.^[16]^ This subdivision according to the number of inhabitants was chosen after checking the type of industrial activity within the different populations of the community with data provided by Spanish National Institute of Statistics. We verified that populations with less than 20,000 inhabitants had a mainly agricultural and livestock activity, whereas those with more than 20,000 inhabitants were urban centers whose main activity was industrial and service sectors.

### 2.2 Methods

The established eligibility criteria were: age under 15 years; a primary diagnosis of childhood cancer with microscopic cancer confirmation defined by the International Classification of Childhood Cancer, third edition (ICCC-3), including benign tumors of CNS; the children must live in CL at time of diagnosis; and must be diagnosed since January 1, 2003 to December 31, 2014.

The collected data included age at diagnosis, sex, personal history of previous pathologies, type and subtype of childhood cancer, inhabited locality, population area (urban or rural), age at death, and time of survival as main variables of interest.

Childhood Cancer Registry of CL (Spain) and the Spanish Childhood Cancer Registry of the Pediatric Oncohematollogy Spanish Society called RETI-SEHOP in Spanish were the main sources of information to capture new cases diagnosed during study period.^[18]^ These sources provide data of age at diagnosis, sex, type and subtype of childhood cancer, inhabited locality, locating the inhabited population through the zip code, age at death, and time of survival. Survival data were confirmed as of December 31, 2015 through information provided by the National Institute of Deaths.

The Childhood Cancer Registry of CL (Spain) is a population-based registry officially created in 2010,^[19]^ but with data collected that cover the entire pediatric population since 2003, with active case searching in multiple sources (not including death certificates).^[20,21]^ Cases were registered by diagnostic group, as defined by the International Classification of Childhood Cancer, third edition (ICCC-3) and by checking the clinical-pathological diagnosis with the International Classification of Diseases for Oncology, third edition (ICD-O-3). Hematological malignancies, malignant and benign tumors of the CNS, and all other malignant solid tumors were selected, following European Network of Cancer Registries recommendations.

Duplication of data was avoided by comparing the database of the population registry of childhood tumors of CL with that of the RETI-SEHOP.

Incidence rates were calculated per million person-years. ASRw were calculated using the world standard population for the age groups under 15 years and by sex to compare with the data published in the literature. Sex ratio (male/female) (SR) is provided to analyze the predominance of each sex according to the tumor type. Ages were grouped based on standard categories: <1; 1 to 4; 5 to 9; 10 to 14, and used for data summary. For rate calculations the groups formed by age were: 0 to 4; 5 to 9; and 10 to 14. Comparative incidence figures (CIFs, ratio of the ASRw) were calculated to compare ASRW incidence rates between rural and urban areas by tumor and age group using EpiDAT 4.0 program.

We used SPSS v23.0 program to calculate the 1, 3, and 5-year overall survivals with the Kaplan-Meier method and to comparing survival curves of 2 groups using the log-rank test. Vital status (alive or dead) at the closing date of the survival study (December 31, 2015) was obtained by active follow-up. No children were lost to follow-up at the date of last contact.

Quantitative variables are expressed as median, with 25th (p25) and 75th (p75) percentiles. *P* value <.05 was considered as significant result. Bivariate comparisons of continuous variables were performed using the Mann-Whitney test. Bivariate comparisons of percentages were performed using the chi-square test. Confidence intervals (CIs) were calculated at 95%.

### 2.3 Ethical approval

The study had the approval of the General Public Health Direction of CL Government, following specific regulations for population records.^[22]^ Personal information data were manipulated according to Spanish data protection rules (Organic Law 15/1999 on December 13).

## 3 Results

For the 12-year period of the study, 615 childhood cancer cases were diagnosed—346 male (56.26%) and 269 female (43.74%). By age groups, 237 cases (38.5%) were registered aged 0 to 4 years (21.1% less than 1 year old), 173 children aged 5 to 9 years (28.13%), and the rest, 205 cases, aged 10 to 14 years (33.3%). In all, 268 children lived in rural areas (43.58%) and 347 in urban areas (56.42%).

None of them had relevant previous pathological backgrounds, and all had lived in our region since birth. Socioeconomic status was similar between the groups compared.

Data quality indicators were: 570 (92.68%) histologically verified; 60 (9.75%) unspecified cases (ICCC categories Ie, IIe, IIIf, VIc, VIIc, VIIIe, XIIb, Xe [M-8000-M8004 only] and XIf [C76 to C80.9 only]); 4 (0.65%) cancer cases of unknown primary origin. For survival data, the median duration of follow-up was 5.88 years (p25 = 2.33, p75 = 8.50).

Overall age-adjusted incidence for 2003 to 2014 was 176.6 cases per million, with incidence being highest at age 0 to 1 year (235.6), lower at age 1 to 4 years (201), and lower still at age 5 to 9 (146.4) and 10 to 14 years (165.6) (Table 1). Hematological tumors accounted for 40.8% and solid tumors for 59.2% of total childhood cancers. Leukemias were most common (ARSw: 44.46, for ALL 37.71 and for ML 5.92), CNS tumors second (ASRw: 39.98), and lymphomas third (ASRw: 25.02, for Hodgkin lymphoma 11.27, for non-Hodgkin lymphomas with Burkitt lymphomas included 12.44, and for Burkitt lymphoma 7.17) (Table 1). ASRw for all neoplasms was 199.81/million (95% CI 179.9–222.65) for boys (n = 347) and 160.13/million (95% CI 141.26–180.93) for girls (n = 268; *P* = .012); for leukemias, it was 40.65 (95% CI 31.71–51.53) for boys (n = 73) and 49.8 (95% CI 39.46–62.12) for girls (n = 82; *P* = .33). Sex ratio was 1.29 for all childhood cancer (Table 1) and 0.89 for leukemias (0.55 at age 0–4 years, 0.93 at age 5–9 years, and 1.59 at age 10–14 years). Total leukemia cases for subtypes, and rural and urban areas are shown in Table 2. For all cancers, median age at diagnosis was 6.3 years old (p25 = 3.0, p75 = 11.0), and between rural and urban areas there was no difference by age (*P* = .38) and sex (*P* = .32).

**Table 1:**
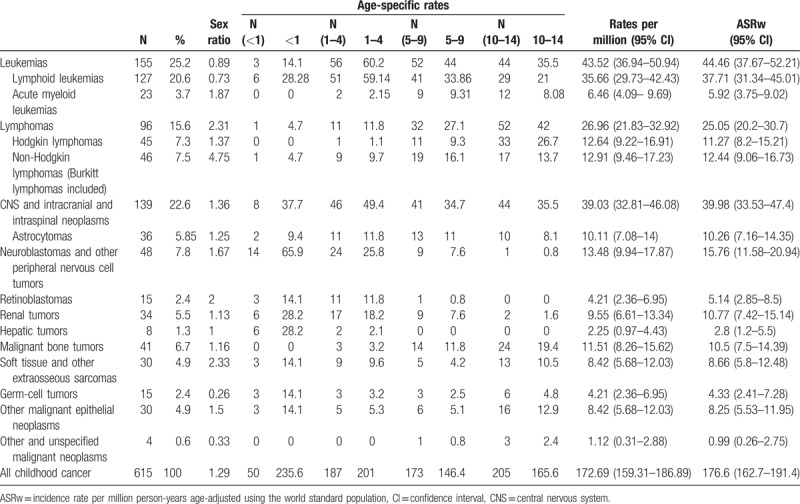
Childhood cancer incidence by diagnostics groups of the International Classification of Childhood Cancer—third edition.

**Table 2:**
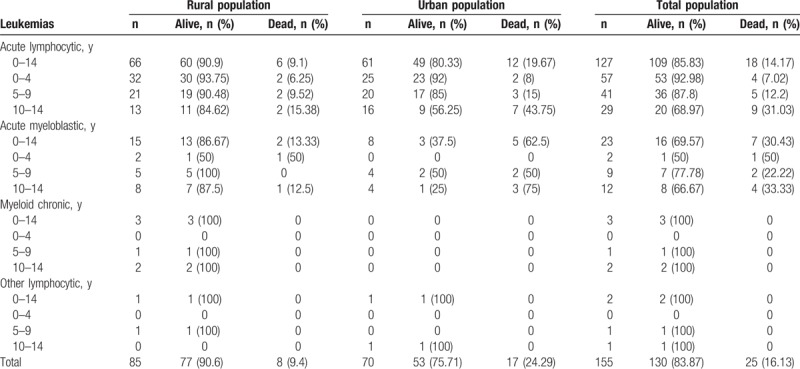
Leukemia case distribution by rural and urban areas.

Overall incidence rates in rural and urban areas for all cancer, leukemias, lymphomas, CNS, and all other solid tumors grouped are presented in Table 3. For leukemias, significant differences in incidence were found, and were higher in rural areas (51.08) than in urban areas (33.65; *P* = .018). Incidence analysis by age showed higher rural leukemia incidence at age 0 to 4 years (67.13) than in urban areas (39.32; *P* = .05), and with no significant differences at age 5 to 9 years and 10 to 14 years. There were no differences between rural and urban areas in lymphomas, CNS and all other malignant solid tumors grouped (excluding CNS) (Table 3).

**Table 3:**
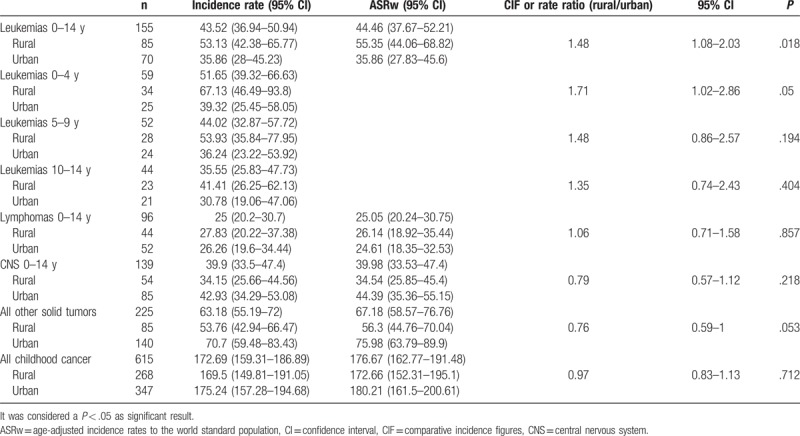
Childhood cancer incidence rate by rural and urban areas.

Survival data are presented in Table 4. For all cancers, 5-year overall survival was 84% (95% CI 79.6–87.5). There were no survival differences by sex for all cancers (*P* = .976), leukemias (*P* = .591), lymphomas (*P* = .933), CNS (*P* = .613), and all other solid tumors grouped (*P* = .445). For the 2 populations, overall survival was 88% (95% CI 83.4–91.4) for rural areas and 80% (95% CI 75.7–83.6) for urban areas (*P* = .033). By etiological groups, although 5-year overall survival was greater in rural than urban areas for all of them, these differences only were significant in leukemias (90% in rural areas and 76% in urban areas; *P* = .01). For this reason, when we analyzed leukemia by age groups, only for children of aged 10 to 14 years, there were significant survival differences, with a rate of 86% (95% CI 64.8–94.9) in rural areas compared with 51% (95% CI 28.3–69.8) in urban areas (*P* = .007) (Table 4). There were no differences in LMA percentages between rural (17.64%) and urban areas (11.42%; *P* = .333) (Table 2).

**Table 4:**
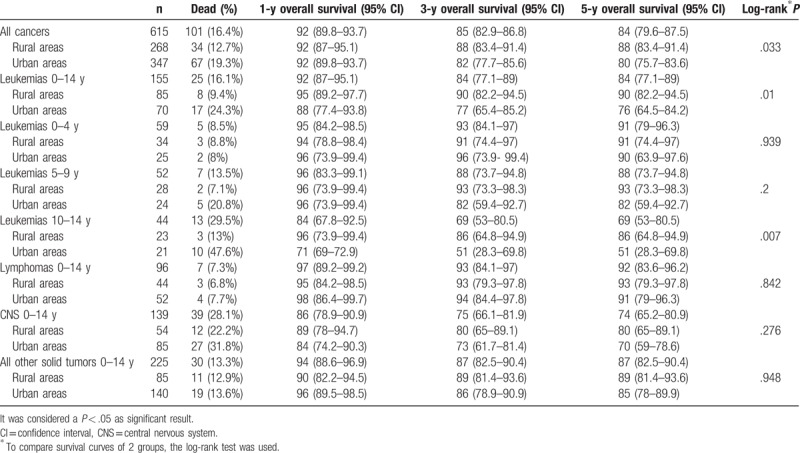
Overall survival by diagnosis groups and age groups.

## 4 Discussion

The childhood cancer incidence pattern in CL was similar to that in Southern Europe and Spain,^[1,23,24]^ although for all tumors combined, lymphomas, and CNS, it was slightly higher.^[23,25]^ For CL childhood cancer we observed 176.6 cases/million, similar to other southern European regions such as Malta (178 cases/million) or Croatia, and Italy (168 cases/million),^[24]^ and slightly higher than other Spanish regions (155.8 cases/million).^[1,25]^ The incidence of lymphoma in CL was very high (25.02 cases/million), as has been reported in the Mediterranean region, and was much higher for Burkitt lymphoma (7.17 cases/million)^[24,25]^—both rates being slightly higher than other Spanish regions and similar to data for Italy.^[1]^ We report a high incidence of all CNS tumors in CL, as has been noted in high-income countries, associated with the extensive availability of diagnostic facilities.

Our incidence rate for all cancers was slightly higher in boys than in girls,^[1,23,24]^ and by diagnostic group, the germ cell and gonadal tumor sex ratio were more common in girls than in boys, as has been widely reported.^[1,13,23–25]^ In contrast to other reports, the leukemia sex ratio in our population was slightly higher in girls in the 0 to 14-year age group (SR = 0.89) and much higher at age 0 to 4 years (SR = 0.55), and was only predominant in boys in the 10 to 14-year age group (SR = 1.59). Because our number of cases is small, female predominance in the 0 to 4-year-old leukemia group might simply be due to chance, yet if confirmed over time, would require further exploration and surveillance,^[26]^ for example, by seeking differences by sex in polymorphisms of childhood acute leukemia susceptibility, which might modify the age of onset in our population.

Because cancer registries do not usually reflect this variable, few studies analyze differences in incidence and survival between urban and rural areas. In addition, another difficulty when comparing results is that the criteria for designating territories as *rural* are not standardized. In CL, rural areas are characterized by a low population density and the importance of agricultural activity, and the urban population by services and industrial activities, but with urban centers of less than 300,000 inhabitants and little traffic pollution. Taking into account these population characteristics, we report higher leukemia incidence in rural areas, due particularly to a higher leukemia incidence at age 0–4 years.^[27]^ Some case control studies of childhood leukemias report increased risk in urban areas caused by residential proximity to industrial and urban sites.^[12,15,28,29]^ However, many authors report a link between exposure to pesticides, either prenatal or early in life, and childhood leukemia.^[12,15,30]^ Parents working in farming, and thus greater childhood exposure to pesticides in rural areas than in urban areas, seems to have been proven as a cause of increased incidence.^[31]^ One mortality-based study of childhood leukemia found a significant increase in leukemia incidence in rural areas linked to agricultural activities and exposure to pesticides.^[12,15]^ Taking into account the environmental factors associated with childhood leukemia, and depending on demographic characteristics, it is possible that traffic pollution, predominant industrial or farming activities, and also certain environmental factors, may influence some populations more whereas other populations may be influenced by other factors.

For all cancers, observed 5-year overall survival was 84% (95% CI 79.6–87.5), for a comparable time period,^[23–25,32]^ similar to most European countries such as the whole of Spain (78%), Italy and the Great Britain (82%), Germany (84%), Austria (85.9%), and the USA (83%), but higher than Eastern Europe (60%–77%).^[23–25,32]^ There were no survival differences by sex for all cancer, leukemias, lymphomas, CNS, and all other solid tumors grouped, as reported by other authors.^[23–25,32]^ By age groups, we found similar survival rates to other contributions. Nevertheless, by population, we report significant survival differences for leukemias and overall childhood cancer between areas with better survival in rural areas in our population. This finding, not previously reported, due to the still small number of cases, must be confirmed over time in our population, and, if confirmed, would require justification by investigating prognostic differences between rural and urban leukemias in children.

Some publications defend the predisposition to childhood cancer in relation to infections, such as the development of leukemias and lymphomas in North Africa in relation to EBV or HIV infections,^[33,34]^ the predisposition being different depending on the availability of healthcare, being smaller in rural areas. This is not happening in our country, because we have an equitable health system.

The limitations of our study include that the division into rural and urban areas according to the number of inhabitants can vary between different world regions depending on the population distribution. Therefore, it is important not only to take into account the territorial distribution plan of each region, but also the type of work activity performed in each of them. Another limitation of our study is the small number of cases in some groups when we subdivide by type of childhood cancer, age, and poblational area (rural or urban). This made some of the differences observed between groups do not reach statistical significance such as the greater 5-year overall survival observed for CNS tumors, lymphomas, and other solid tumors in rural areas.

## 5 Conclusions

In conclusion, this investigation is the first population study to describe childhood cancer incidence and survival in CL (Spain) and 1 of the few cohort studies that analyze childhood cancer by population areas with data from population-based registries, providing high quality to the conclusions given.^[23–25,32]^ As in the publications discussed above, our study demonstrates a higher childhood cancer incidence in our region, with a general predominance of cancer in boys, similar to development countries. Further studies are needed to explain the predominant female sex ratio in leukemias at 0 to 4 years and the incidence differences observed in leukemias at 0 to 14 and 0 to 4-year age group, and also survival differences observed in leukemias at 0 to 14-year age group between rural and urban areas in our population. We consider this study the point of start of other epidemiological investigations in childhood cancer which include genetic, epigenetic, and environmental factors, analyzing risk factors in urban and rural areas that could explain the facts observed not only in our region but also in the world.

## Acknowledgments

The authors thank the collaboration of Childhood Cancer Registry of Castilla y León and Spanish Childhood Cancer Registry of the Pediatric Oncohematollogy Spanish Society (RETI-SEHOP).

## Author contributions

**Conceptualization:** Hermenegildo González García, Rebeca Garrote Molpeceres, Pilar Gutierréz Meléndez, María Asunción Pino Vázquez.

**Data curation:** Rebeca Garrote Molpeceres, Pilar Gutierréz Meléndez, Raquel Herraiz Cristobal.

**Formal analysis:** Hermenegildo González García, Rebeca Garrote Molpeceres, Elena Urbaneja Rodríguez.

**Funding acquisition:** Elena Urbaneja Rodríguez, Raquel Herraiz Cristobal.

**Investigation:** Hermenegildo González García, Rebeca Garrote Molpeceres, Elena Urbaneja Rodríguez, Raquel Herraiz Cristobal.

**Methodology:** Hermenegildo González García, Rebeca Garrote Molpeceres, Elena Urbaneja Rodríguez.

**Project administration:** Pilar Gutierréz Meléndez, Raquel Herraiz Cristobal, María Asunción Pino Vázquez.

**Supervision:** Hermenegildo González García, Pilar Gutierréz Meléndez, María Asunción Pino Vázquez.

**Validation:** Hermenegildo González García, Rebeca Garrote Molpeceres, Elena Urbaneja Rodríguez, Pilar Gutierréz Meléndez, María Asunción Pino Vázquez.

**Writing – original draft:** Hermenegildo González García, Rebeca Garrote Molpeceres, María Asunción Pino Vázquez.

**Writing – review & editing:** Hermenegildo González García, Rebeca Garrote Molpeceres.
